# Carbonate compensation depth drives abyssal biogeography in the northeast Pacific

**DOI:** 10.1038/s41559-023-02122-9

**Published:** 2023-07-24

**Authors:** Erik Simon-Lledó, Diva J. Amon, Guadalupe Bribiesca‐Contreras, Daphne Cuvelier, Jennifer M. Durden, Sofia P. Ramalho, Katja Uhlenkott, Pedro Martinez Arbizu, Noëlie Benoist, Jonathan Copley, Thomas G. Dahlgren, Adrian G. Glover, Bethany Fleming, Tammy Horton, Se-Jong Ju, Alejandra Mejía-Saenz, Kirsty McQuaid, Ellen Pape, Chailinn Park, Craig R. Smith, Daniel O. B. Jones

**Affiliations:** 1 National Oceanography Centre, Southampton, UK; 2 SpeSeas, D’Abadie, Trinidad and Tobago; 3 Marine Science Institute, University of California, Santa Barbara, CA USA; 4 Natural History Museum, London, UK; 5 Institute of Marine Sciences—Okeanos, University of the Azores, Horta, Portugal; 6 Centre for Environmental and Marine Studies & Department of Biology, University of Aveiro, Aveiro, Portugal; 7 German Centre for Marine Biodiversity Research, Senckenberg am Meer, Wilhelmshaven, Germany; 8 Institute for Biology and Environmental Sciences, Carl von Ossietzky University, Oldenburg, Germany; 9 Ocean & Earth Science, University of Southampton, Southampton, UK; 10 NORCE Climate and Environment, Bergen, Norway; 11 Department of Marine Sciences, University of Gothenburg, Göteborg, Sweden; 12 Korea Institute of Ocean Science and Technology, Busan, South Korea; 13 Ocean Science Major, University of Science and Technology, Daejeon, South Korea; 14 University of Plymouth, Plymouth, UK; 15 Marine Biology Research Group, Ghent University, Ghent, Belgium; 16 Department of Oceanography, University of Hawai’i at Manoa, Honolulu, HI USA

**Keywords:** Biogeography, Marine biology, Macroecology

## Abstract

Abyssal seafloor communities cover more than 60% of Earth’s surface. Despite their great size, abyssal plains extend across modest environmental gradients compared to other marine ecosystems. However, little is known about the patterns and processes regulating biodiversity or potentially delimiting biogeographical boundaries at regional scales in the abyss. Improved macroecological understanding of remote abyssal environments is urgent as threats of widespread anthropogenic disturbance grow in the deep ocean. Here, we use a new, basin-scale dataset to show the existence of clear regional zonation in abyssal communities across the 5,000 km span of the Clarion–Clipperton Zone (northeast Pacific), an area targeted for deep-sea mining. We found two pronounced biogeographic provinces, deep and shallow-abyssal, separated by a transition zone between 4,300 and 4,800 m depth. Surprisingly, species richness was maintained across this boundary by phylum-level taxonomic replacements. These regional transitions are probably related to calcium carbonate saturation boundaries as taxa dependent on calcium carbonate structures, such as shelled molluscs, appear restricted to the shallower province. Our results suggest geochemical and climatic forcing on distributions of abyssal populations over large spatial scales and provide a potential paradigm for deep-sea macroecology, opening a new basis for regional-scale biodiversity research and conservation strategies in Earth’s largest biome.

## Abstract (web summary)

Analysing seabed imagery data spanning 5,000 km and >50,000 megafaunal specimens across the Clarion–Clipperton Zone abyssal plain in the northeast Pacific, the authors identify two distinct biogeographical provinces in which taxa that are dependent on calcium carbonate structures are restricted to depths above 4,300–4,880 m.

## Main

The abyssal seabed lies between water depths of 3,000 and 6,000 m (ref. ^1^), representing most of the Earth’s surface and harbouring some of its most extensive but least explored ecosystems. This abyssal seabed consists of plains and hills that extend across ocean basins, interspersed with seamounts and subdivided by mid-ocean ridges, ocean trenches and fracture zones^1^. Lacking sunlight, energy in this environment is highly limited, with detrital particles sinking from surface waters providing the main source of food^2^, while temperatures are steady at 0.5–3.0 °C (ref. ^3^) and bottom currents are generally low (0–0.25 m s^−1^) (ref. ^4^). As a result, abyssal benthic communities typically exhibit low abundance and biomass compared with other marine environments^5^ but they support high species richness at regional to landscape scales^6,7^ and play an active role in the cycling of carbon^8^. Abyssal habitats are therefore considered to be reservoirs for biodiversity^7,9^ and sources of important ecosystem services^2,10^. Despite their remoteness and as a consequence of being energy-restricted and environmentally stable, abyssal habitats are also expected to be highly vulnerable to anthropogenic disturbances including climate change^11,12^ and emerging industrial activities such as polymetallic nodule mining^13,14^. To adequately conserve the biodiversity and services of abyssal seabed ecosystems, it is critical to consider and protect their full range of habitats and communities, which requires elucidating the patterns and processes controlling the distribution of abyssal populations over large, regional scales.

Macroecological studies assessing spatial variation on regional to global scales in the deep ocean^15–18^ have revealed that faunal body size and abundance gradually decline with decreasing energy availability along gradients such as increasing depth^5,19^ or latitude^16,20^. These relationships are thought to constrain higher-order community structure and function^21^, for example, reducing the diversity of larger-sized taxa with depth^22^. Predicted declines in species richness with depth are consistent with checklist-based analyses of large marine databases^23^. Overall, results from large-scale studies in the deep ocean confirm clear variation between abyssal and (shallower) bathyal seafloor ecosystems^16,19,22^, which encompass a variety of relatively well-known habitats, between 1,000 and 3,000 m depth, including continental slopes, submarine canyons and coral mounds. Consequently, while biogeographic provinces proposed for the deep ocean readily distinguish bathyal and abyssal provinces within oceanic basins^24^, potential variation within abyssal regions remains largely unresolved a decade after provincial boundaries based on surrogate environmental data were postulated^24^, since large-scale empirical studies (for example, bottom-up assessments) are lacking for remote abyssal ecosystems. As such, the processes regulating alpha- and beta-diversity across the scale of a seemingly connected abyssal plain remain unclear, particularly for the largest size-class of seafloor organisms, megafauna (animals >10 mm in maximum dimension). Megafauna are a conspicuous component of the abyssal benthos and a common target for investigation in deep-sea spatial ecology^13,25–27^ because occurrences of these taxa can be determined across large spatial scales from seabed imagery^28^ collected by deep-sea robots^29^.

**Fig. 1:**
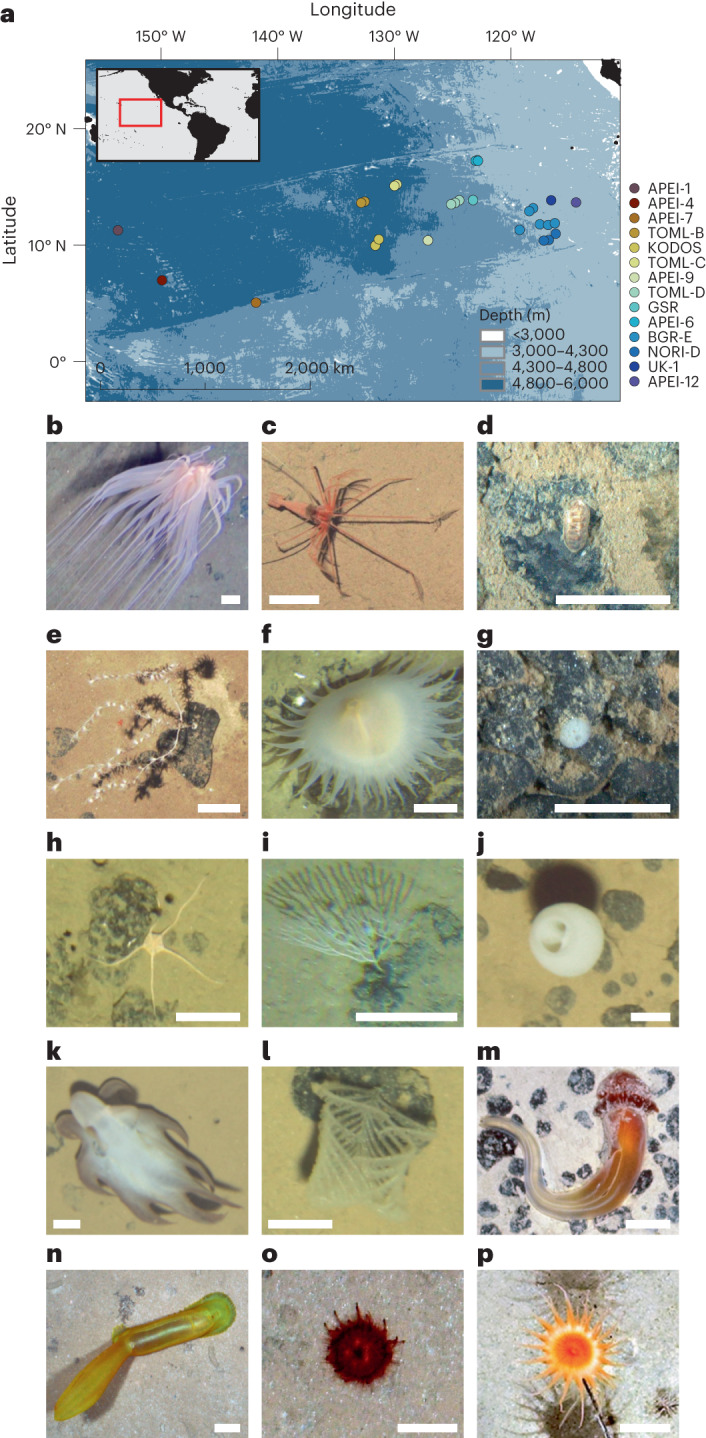
Study region in the northeast Pacific basin and examples of abyssal benthic megafauna typically encountered at different depth ranges. **a**, Map of study locations surveyed using deep-sea robots (ROVs and AUVs). Points indicate locations (depths 3,900–5,300 m) where data from seabed imagery studies were collated from, aligned and reanalysed using standardized methodology for homogenous detectability and taxonomic identification of invertebrate benthic megafauna (animals >10 mm). The colour of the points follows a consistent scheme used to differentiate each site in the other figures. **b**–**p**, Examples of abyssal Pacific metazoan megafauna morphotypes (including depth, site and code in standardized catalogue), ordered by depth. **b**, *Relicanthus daphneae* sp. inc. (3,914 m, APEI-12, REL_001). **c**, *Bathystylodactylus echninus* (4,005 m, APEI-6. DEC_009). **d**, *Leptochiton* sp. indet. (4,205 m, NORI-D, MOL_002). **e**, *Bathygorgia profunda* sp. inc. (4,050 m, APEI-6, ALC_004), growing attached to a fossilized *Otodus megalodon* shark tooth. **f**, Sicyonidae gen. indet. (4,247 m, BGR-E, ACT_002). **g**, *Thenea* sp. indet. (4,190 m, NORI-D, DES_021). **h**, *Ophiosphalma glabrum* sp. inc. (4,621 m, TOML-D, OPH_010). **i**, Bifaxariidae gen. indet. (4,210 m. BGR-E, BRY_012). **j**, *Hyalonema clarioni* sp. inc. (4,848 m, TOML-C, HEX_002). **k**, *Grimpoteuthis* sp. indet. (4,959 m, TOML-C, MOL_008). **l**, *Abyssopathes lyra* (4,770 m, TOML-B, ANT_002). **m**, *Tergivelum* sp. indet. (5,019 m, KODOS, HEM_005). **n**, *Psychropotes* sp. indet. (5,007 m, APEI-4, HOL_047). **o**, *Kamptosoma abyssale* sp. inc. (5,240 m. APEI-1, URC_010). **p**, Actinostolidae gen. indet. (4,620 m, APEI-9, ACT_088).

To address these knowledge gaps, we compiled a new basin-scale standardized dataset to investigate patterns of abundance and diversity in benthic megafaunal communities across the Clarion–Clipperton Zone (CCZ, northeast Pacific). The CCZ spans ~6 million km^2^ of abyssal plain in areas beyond national jurisdiction between the Exclusive Economic Zones of Kiribati and Mexico (Fig. 1a) and is the largest area in the world currently in exploration phase for mineral mining^30,31^. We selected data from 12 expeditions using comparable seabed imaging approaches to study invertebrate benthic megafauna distribution patterns across the CCZ. We collated, reanalysed, aligned and taxonomically standardized seabed imagery data from these studies including three new sites covering a total of >150,000 m^2^ of seabed, which represents an area two orders of magnitude greater than that commonly assessed in studies of abyssal megafauna (for example, ref. ^27,32^, <5,000 m^2^). Our dataset spans 5,000 km across the CCZ (28 geographical locations) encompassing >50,000 megafaunal specimens from 411 morphotypes (morphologically identifiable taxonomic units^33^) in 13 phyla, enabling investigation of ecological gradients (for example, with latitude, depth or food supply) in seabed communities across the region to develop key understanding of macroecological patterns in Earth’s largest biome.

## Results and discussion

### Macroecological patterns in the northeast Pacific abyss

**Fig. 2:**
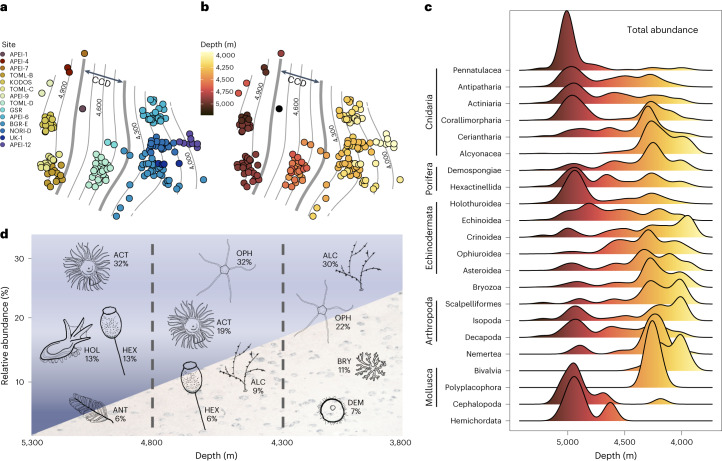
Variations in the taxonomic composition of invertebrate megafaunal communities demarking biogeographic provinces within the northeast Pacific abyss. **a**,**b**, Two-dimensional representation of multidimensional scaling analyses, depicting assemblage Bray–Curtis dissimilarity rates (distance) calculated between 161 independent community samples (containing 200 specimens identified to morphotype level per sample) across 28 geographical locations. **a**,**b**, Sample point colour coding: study site of each sample location (**a**) or mean depth at sample location (**b**). Arrow depicting the spatial extent of the carbonate compensation depth (CCD) across the northeast Pacific. Isotropic contour lines (fitted using GAMs) represent rough approximates of depth-range bins to aid visualization of patterns. **c**, Ridgeline plots outlining the geometric distribution of total abundance along depth for the dominant taxonomic groups in the abyssal CCZ megabenthos. On the *y* axis, frequency distribution relative to all the specimen occurrences sampled per group; colour, depth range (as depicted in **b**). **d**, Top four dominant taxonomic groups within the communities of different depth ranges (background seabed illustrating depth variation exaggerated across the region). ACT, Actiniaria; HOL, Holothuroidea; HEX, Hexactinellida; ANT, Antipatharia; ALC, Alcyonacea; OPH, Ophiuroidea; BRY, Bryozoa; and DEM, Demospongia. Note that depth was plotted decreasing from left to right, that is, west to east, to mirror the approximate spatial pattern across the CCZ.

Our analyses reveal the existence of a clear depth zonation demarking an evident boundary between biogeographic provinces across the northeast Pacific, with distinct shallow and deep-abyssal faunas and an intermediate transition zone. Regional benthic community structure at higher taxonomic levels exhibited a more pronounced relationship to depth (Fig. 2b) than other environmental gradients (for example, Supplementary Fig. 1) while food particle supply appears to be important at more intermediate, sub-regional, spatial scales (see below). Our results show distinct communities but incomplete taxonomic replacement in the presence and relative abundance of seabed taxa across the depth range studied, both within and between phyla. The most remarkable shifts were as follows: (1) soft corals (Alcyonacea) numerically dominated at shallow-abyssal depths (mean density ~2,500 individuals ha^−1^) but decreased in abundance by over an order of magnitude below 4,300 m (mean density ~100 ind ha^−1^), becoming virtually absent below 4,800 m (mean density <10 ind ha^−1^); (2) brittle stars (Ophiuroidea) were most abundant at shallow-abyssal depths (mean density ~2,000 ind ha^−1^), numerically dominated assemblages at intermediate depths (4,300–4,800 m; mean density ~1,500 ind ha^−1^) but decreased to low abundances below 4,800 m (mean density <40 ind ha^−1^); (3) anemones (Actiniaria) consistently increased in relative abundance with depth, first replacing soft corals as the dominant Cnidaria group below 4,300 m and then replacing brittle stars as the most dominant group in assemblages below 4,800 m (Fig. 2d); (4) sea cucumbers (Holothuroidea) largely increased in relative abundance with depth, replacing brittle stars as the most abundant Echinodermata group below 4,800 m (Fig. 2d); (5) shelled molluscs (for example, Bivalvia, Gastropoda and Polyplacophora), relatively abundant above 4,400 m (mean density ~300 ind ha^−1^), were absent below 4,400 m, where cirrate octopuses (Cephalopoda) became the only molluscan group in megabenthic assemblages. Changes in dominant taxa at the class or phylum level (Fig. 2c) clearly delimited two distinct abyssal assemblages (Fig. 2d): a shallow community (3,800–4,300 m) and a deep community (4,800–5,300 m). These were separated by an intermediate or transitional assemblage (4,300–4,800 m), which contained elements of both communities. No major taxonomic groups were restricted to the transitional zone (Fig. 2c).

The zonation pattern was also evident at lower taxonomic levels in the 411 invertebrate morphotypes recognized in the data. For example, from the 277 morphotypes found in the deep province, 60 (41 rare, that is <5 occurrences) were found exclusively in this deep zone, while 64 morphotypes (45 rare) out of a total of 283 were found exclusively in the shallow province. However, only 25 morphotypes (23 rare) from a total of 264 were found exclusively in the transitional zone. From the total of 175 (14 rare) morphotypes that were found in both shallow and deep communities, only two were within the ten most dominant taxa in both the shallow province (first- and third-most abundant) and in the deep province (nineth- and tenth-most abundant; Supplementary Table 2): the brittle star *Ophiosphalma glabrum* (Fig. 1h; well-studied in the eastern CCZ^34^) and *Thenea* sp. indet. (Fig. 1g), an undescribed species of nodule-dwelling sponge.

The nature of the taxonomic shifts between provinces and the depth range at which these occur suggests that the carbonate compensation depth (CCD) plays a key role in the depth zonation we encountered. The CCD lies close to the seafloor depth in the northeast Pacific^35^, deepening north to south from ∼4,400 to ∼4,800 m (ref. ^36^). Below the CCD, calcium carbonate (CaCO_3_) is undersaturated, which can energetically constrain the development and distribution of species highly dependent on carbonate structures such as in calcareous Foraminifera, a ubiquitous protozoan group showing clear depth zonation at intermediate scales in the abyss^37,38^. It is these groups, probably depending on CaCO_3_ structures, that exhibit the greatest variation in relative abundance or even presence with depth in our analysis and thereby contribute most to regional beta-diversity. For example, shelled molluscs (requiring biomineralization of CaCO_3_ for shell development^39^) were absent below 4,400 m and soft corals (with scleroblast cells requiring CaCO_3_ to produce sclerites^40^) and bryozoans (most species dependent on CaCO_3_ exoskeletons^41^) were replaced by ‘soft’ anemones (Actiniaria) below 4,300 m. The echinoderm transition from ophiuroids to holothurians below 4,800 m, yielding the dominant taxon of the deep province, could be related to either differing CaCO_3_ requirements or metabolic efficiency, documented across echinoderms^42^. All of these shifts were observed within the depth range (4,400–4,800 m) of the CCD in the northeast Pacific^35,36^. However, our understanding of biogeochemical and metabolic cycles in abyssal ecosystems is still too limited to fully comprehend the specific role of the CCD and calcium calcite saturation, as a biogeographical driver in abyssal metazoans.

**Fig. 3:**
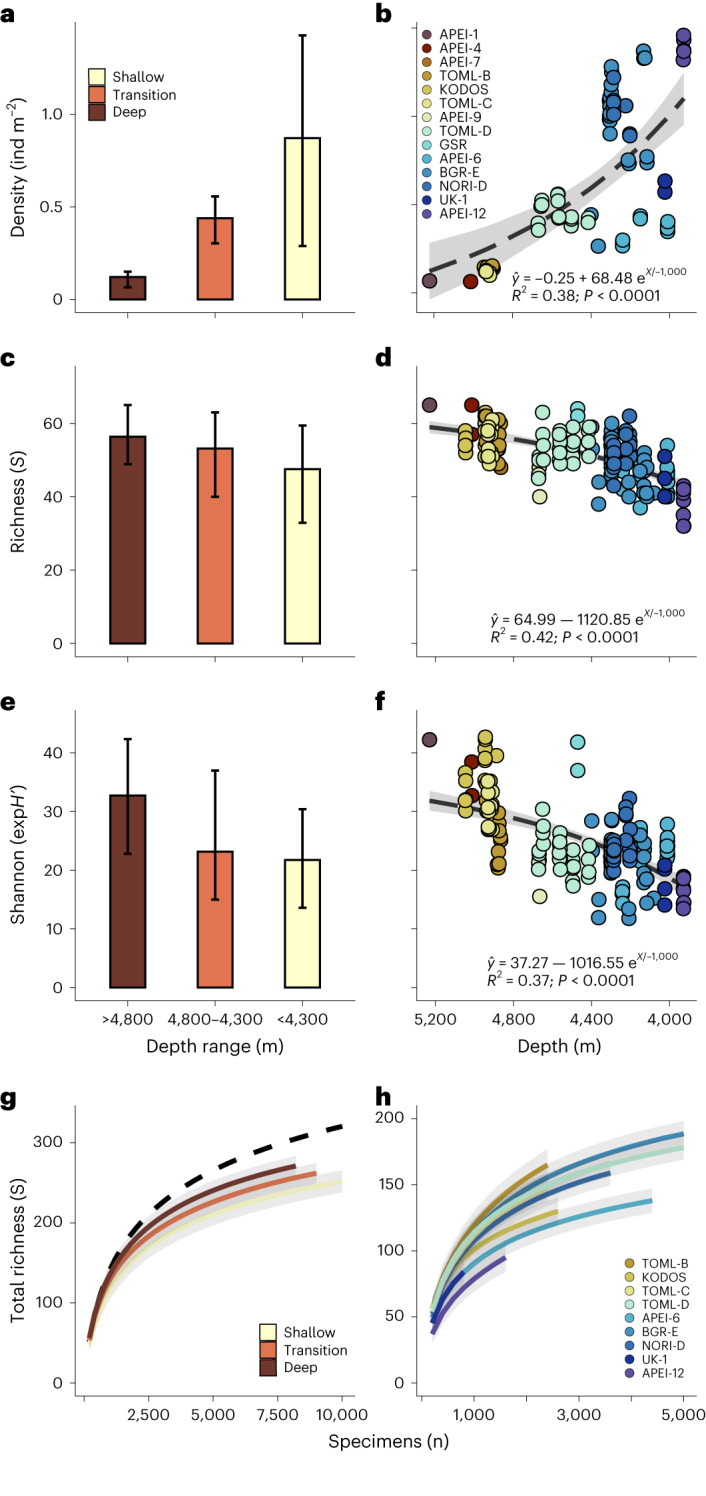
Standing stocks were substantially larger in the shallow than in the deep-abyssal province while biodiversity rates were similar, although slightly increasing with depth, across the northeast Pacific abyss. **a**,**b**, Faunal densities calculated in 84 independent community samples (containing 400–500 specimens) extending across 23 geographical locations. **a**,**b**, Variations in faunal density: between abyssal provinces (*n* = 41 samples in shallow, 27 in transition and 16 in the deep province) (**a**); and across the depth range (*F*_1,82_ = 51.61, *P* = 0.001, 2.81 × 10^−10^) (**b**). **c**–**f**, Diversity estimates calculated in 161 independent community samples (containing 200 specimens identified to morphotype level per sample) extending across 28 geographical locations. **c**,**d**, Variations in morphotype richness (*S*): between abyssal provinces (*n* = 81 samples in shallow, 39 in transition and 41 in the deep province) (**c**); and across the depth range (*F*_1,159_ = 115.4, *P* = 2.2 × 10^−16^) (**d**). **e**,**f**, Variations in the exponential form of Shannon’s diversity index (exp*H’*): between abyssal provinces (*n* = 81 samples in shallow, 39 in transition and 41 in the deep province) (**e**); and across the depth range (*F*_1,159_ = 94.09, *P* = 2.2 × 10^−16^) (**f**). **g**,**h**, Morphotype accumulation curves, showing variations in the total richness sampled: between provinces (dash-line: all data combined) (**g**); and between different sites (including only sites with more than three community samples) (**h**). **a**,**d**,**e**, Mean values (bars) and 95% confidence intervals (error bars) across all the samples in each province. **b**,**d**,**f**, Values calculated for each independent sample (points) and results of linear regression; mean (dashed-line) and 95% confidence intervals (shallowing). **g**,**h**, Mean values across 100 randomizations (lines) and 95% confidence intervals (shallowing). Note depth was plotted throughout decreasing from left to right, west to east, to mirror the approximate spatial pattern across the CCZ.

Remarkably, despite the shifts in benthic community structure between provinces, diversity rates were relatively similar across biogeographic boundaries (Fig. 3c). Richness (Hill’s *q* = 0) ranged between 32 and 62 morphotypes per 200 specimens in the shallow-abyssal province and between 48 and 63 in the deep one but we found no evidence of a difference in richness across the three depth ranges (overlapping 95% confidence intervals; Fig. 3c). We also found little evidence of variations between deep, transition and shallow assemblages in Shannon diversity (Hill’s *q* = 1; Fig. 3e), a metric more sensitive to species evenness^43^. However, we found very strong evidence of an increase in both taxa richness (*F*_1,159_ = 115.4, *P* < 0.001) and Shannon diversity (*F*_1,159_ = 94.09, *P* < 0.001) with increasing depth (Fig. 3d,f). This reflects a higher evenness of taxa in the deep province. For instance, the ten most abundant morphotypes in the shallow and intermediate depth ranges encompassed ~60% of the total abundance sampled in these areas, whereas below 4,800 m, the ten most abundant taxa represented only ~40% of all fauna (Supplementary Table 2). An increase in evenness across ecological communities is often related to a decrease in surface productivity^44^ and, in abyssal areas, greater flux of sinking food is usually found in shallower locations nearer to surface primary production^2,8^. This is generally the case in the northeast Pacific region^45^ and seems a plausible driver for the higher evenness observed at regional scale in the deep province, particularly as our sampling approach was specimen-rarefied. The higher evenness of the deep community was also evident in species accumulation patterns (that is, steeper initial accumulation; Fig. 3g). However, with increasing sampling effort, estimated total richness of the deep province remained consistently higher than the shallower communities, suggesting that both biodiversity components (richness and evenness) were not only maintained but slightly enhanced, with increasing depth.

Our results distinguish two abyssal biogeographic provinces with remarkably distinct benthic community features within a region (northeast Pacific) where a previous top-down environmental assessment identified the potential for two abyssal provinces^24^. We found very strong evidence of different faunal densities between the deep and the shallow provinces (Fig. 3a), with megabenthic standing stocks largely decreasing with depth (*F*_1,82_ = 51.61, *P* < 0.001, Fig. 3b). As was expected from local megafaunal studies^25–27^, density (range 0.06–1.46 ind m^−2^) was up to an order of magnitude larger in the shallow than in the deep province (Fig. 3a). Nonetheless, regional patterns of density within provinces appeared more related to variations in food supply, that is particulate organic carbon (POC) flux, to the seabed (Supplementary Fig. 3a) than depth (Fig. 3b), especially in deposit-feeding fauna (Supplementary Fig. 3b). In particular, the high variability in faunal density across the shallow province (Fig. 3a) probably reflects a latitudinal gradient in POC flux at intermediate scales, with lowest faunal densities in the northern sector (for example, range APEI-6 site: 0.3–0.4 ind m^−2^), increasing towards southeast locations (for example, ranges NORI-D, 0.9–1.2 ind m^−2^; APEI-12, 1.3–1.5 ind m^−2^) in closer proximity to the high primary productivity related to equatorial upwelling^8^, with known capacity to regulate abyssal benthic processes^46^. Similarly, the benthic communities surveyed in northeasternmost locations (for example, APEI-6; ref. ^47^)—for instance, with the largest densities we observed in scavenging megafauna (mostly decapods and isopods; Supplementary Fig. 3d) and an evidently lower total taxonomic richness (Fig. 3h)—were markedly distinct from those in the southeast locations of the shallow province (such as BGR-E, UK-1, APEI-12 and NORI-D; Fig. 2a and Supplementary Fig. 2). These differences could be driven by altered oceanographic conditions over the northeast CCZ, potentially related to the influence of the North Pacific Equatorial Current^48^ and the closer proximity to the continental shelf, which may transport pelagic food falls^49^ more commonly into this sector. Hence, while regional patterns across provinces in abundance, diversity and community composition were clearly zoned by depth over scales of thousands of kilometres, variations at intermediate scales within provinces, over hundreds of kilometres, might be more related to surface productivity gradients^32^ (also at the deep province; for example, Supplementary Fig. 2c) and other environmental (hydrographical) variation between sectors. Overall, this reflects a much higher ecological heterogeneity, at multiple scales, than was previously expected for benthic assemblages across the northeast Pacific abyssal seabed. This overlooked heterogeneity, stemming from geochemical and climatic forcing, has crucial implications for future ecological and macroecological research in abyssal communities and for the success of regional-scale conservation strategies implemented to protect biodiversity in the CCZ^50^ and probably in other abyssal areas targeted by deep-sea mining worldwide^31^.

## Conclusions

The results of this study represent the largest multiphylum assessment of beta-diversity patterns to date conducted across such a vast abyssal seabed extension (>5,000 km span), provide key insights to our view of abyssal ecosystems and biodiversity and challenge some current paradigms in deep-sea macroecology. The maintenance of high taxonomic richness with increasing depth by phylum-level replacements (in presence or dominance) was surprising. Diversity, especially that of the largest-sized fauna, is commonly expected to decrease with depth and nutrient flux in the deep sea^5,19,21,22^, a reduction that is thought to reflect the wider geographic ranges of deep-sea species^23^. But our results suggest that more complex processes may control diversity in the abyss, helping maintain community richness with depth. These interpretations could be affected by the historical unbalance in the sampling conducted in abyssal ecosystems (for example, ~90% of the species living at the CCZ remain undescribed^51^) compared to shallower deep-sea environments^52^. In turn, our results add to the existing evidence^53^ against a source–sink dependency from bathyal to abyssal communities in the Pacific^54^ and stress that, given the wealth of between-phylum species replacement we encountered with increasing depth, assessments including multiple phyla might be best suited to investigate biodiversity and macroecological patterns in abyssal regions.

The presence of such a clear and unexpected boundary zone demarking the limits of provinces has not been previously documented in abyssal metazoans, opening questions for further macroecological research in the deep ocean. We suggest that the CCD may control the location of the environmental boundaries between shallow and deep-abyssal provinces for megafauna, as appears to occur in protozoans (for example, Foraminifera^37,38^), although more physiological research is needed to better understand how CaCO_3_ saturation affects different phyla. Greater understanding of CaCO_3_ saturation as a biodiversity driver is especially important as the CCD is expected to shoal with increased CO_2_ levels in the ocean associated with climate change^55^. In the light of our results, shoaling of the CCD could have much larger implications for abyssal biodiversity than previously expected, especially in regions like the northeast Pacific where the seafloor depths transcend the current CCD^11^. A CCD shoaling of tens of metres in abyssal ecosystems, given their vast area, could lead to shifts in the environmental conditions (for example, from CaCO_3_ saturation to undersaturation) across thousands of km^2^ of seabed worldwide. This could trigger large shifts in community structure and species distributions or even extinctions of highly specialized abyssal taxa, adding to the cumulative impacts of deep-sea mining and other emerging anthropogenic disturbances^56^ that might concur on Earth’s largest biome in the coming years.

## Methods

### Study area

The CCZ is an extensive abyssal plain and hill ecosystem, interspersed by seamount areas^26,57^, covering approximately 6 million km^2^ of seafloor stretching from 5° to 20° N and 115° to 160° W in the northeast Pacific basin (Fig. 1). Important abiotic factors are broadly similar across the northeast Pacific abyssal seafloor, such as low currents, constant bottom-water salinity and temperature^45^. In contrast, there is a gradual increase in water depth from east to west (from 3,700 to 5,500 m; Fig. 1a) resulting from the contraction of older oceanic crust to the west^58^ and a gradient in POC flux to the seabed, generally diminished with depth but largely enhanced towards more productive southeastern waters^59^ (Supplementary Methods). In turn, the CCD (the depth below which the rate of supply of calcium carbonate from the surface is equal to the rate of dissolution^60^) lies close to the seafloor in the northeast Pacific^35^, deepening north to south between 4,400 and 4,800 m (ref. ^36^). Surface sediments in the northeast Pacific are relatively well-oxygenated and generally dominated by either siliciclastic clay and radiolarian ooze in the northern and central parts of the CCZ, or by biogenic calcareous oozes and fine-grained sediments in southern sites^45^. These are supplied by extremely low sedimentation rates, regionally varying between 0.2 and 1.15 cm per 1,000 years^61^. These conditions are thought to promote the (extremely slow) formation of polymetallic nodules^35^, yielding growth rates of ∼1–12 mm per million years in the abyssal Pacific^31^. Nodules can vary in size, shape and abundance^61^ and are extensively distributed across the northeast Pacific^31^ but are not exclusively found there. Vast abyssal nodule field areas are present in the southeast Pacific (for example, Peru Basin), the southwest Pacific (for example, Penrhyn Basin) and in the Central Indian Ocean Basin^31^. Nodule fields form an unusual mosaic habitat, where the hard substratum provided by nodules acts as keystone structure increasing local seabed complexity^62,63^, promoting the occurrence of some of some of the most biodiverse benthic assemblages surveyed in the abyss^25,27^. But the biodiversity of the CCZ is poorly known, for example 90% of the species identified in the region are thought to be new to science^51^ and substantial levels of dark biodiversity have been predicted^34^. In addition, a general lack of standardization of intercalibrated taxonomic standards for the identification of benthic fauna^50^ has historically hampered comparability between biological studies^64^ and thereby the assessment of regional, macroecological patterns across the northeast Pacific basin.

### Data processing

Occurrences of benthic invertebrate metazoans were collated from a range of seabed image surveys collected using comparable sampling methodology across the northeast Pacific (Table 1). To ensure homogenous animal detectability and a minimum sample size per study location, we selected only available imagery obtained using deep-sea robots (remotely operated vehicles, ROVs; and autonomous underwater vehicles, AUVs; and towed-camera platforms) that fulfilled the following criteria: (1) collected between 1 and 4 m above the seabed; (2) well-lit and high resolution (that is, minimum resolution at 2 m above-seabed, 1,280 × 720 pixels); (3) total seabed survey area imaged per location >2,000 m^2^; (4) no overlapping frames included in image-based studies; (5) scalable stills collected vertically facing the seabed (only in data used for density analysis, see below). Imagery data were collected within 14 sites managed by the International Seabed Authority across the CCZ (Table 1 and Fig. 1a); eight mining exploration-licenced sites and six APEIs.

**Table 1:** Seabed imagery data collected across northeast Pacific basin (order west to east) and selected for standardized analysis in the present study. Note that video datasets with high uncertainty of the estimated area surveyed were not used in density analyses and ‘Area sampled’ provided as approximate total linear length of survey transects

Site (locations)	Depth minimum to maximum (m)	Images (*n*)	Area surveyed (m^2^)	Specimens (*n*)	Source
APEI-1 (1)	5,198–5,252	1,250	6,767	457	^26^
APEI-4 (1)	4,999–5,039	1,706	9,529	603	^26^
APEI-7 (1)^a^	4,855–4,873	1,347	7,277	266	^26^
TOML-B (2)	4,419–5,175	6,939	24,980	3,571	^25^
KODOS (2)^a^	4,887–5,065	(video)	> 20 km	4,493	This study^b^
TOML-C (2)	4,817–5,063	8,132	29,275	3,386	^25^
APEI-9 (1)^a^	4,627–4,693	(video)	>8 km	1,057	This study^b^
TOML-D (3)	4,345–4,750	5,612	20,203	8,889	^25^
GSR (1)^a^	4,455–4,480	(video)	>1.2 km	875	^57^
APEI-6 (3)	3,985–4,263	11,555	21,582	7,773	^47,62,63^
BGR-E (6)	4,024–4,423	6,046	15,626	12,910	^86^
NORI-D (3)	4,170–4,320	2,500	5,067	5,160	This study^b^
UK-1 (1)	4,015–4,033	1,355	2,178	1,371	^27,87^
APEI-12 (1)	3,905–3,952	1,102	2,035	2,702	^27,87^

^a^Datasets excluded from density analyses.

^b^Collection methodology provided in Supplementary Methods.

Megafauna specimens >10 mm were detected and counted in imagery reanalysed from the selected surveys (Table 1). Using BIIGLE 2.0 software^65^, animals were identified to the lowest taxonomic level possible (morphotype, typically genus or family level in undescribed species; for example, Fig. 1b–p) in accordance with an abyssal-Pacific standardized megafauna catalogue^66^ developed during a range of scientific workshops, in collaboration with taxonomic experts (see Acknowledgements), and by reference to existing literature^67–69^—though developed before the major higher taxonomic revision recently undergone by Octocorallia^70^. The catalogue follows the ref. ^33^ open nomenclature to report the taxonomic resolution reached in each morphotype but all taxa identified from the catalogue (411 morphotypes in this study) were deemed as sufficiently different morphologically by taxonomic experts to be confidently considered separate species. Specimens with uncertain classification at the morphotype level (~30% of all records) but identifiable to a certain higher taxonomical level (like phylum or class) were retained only for density analyses. Taxa living in a closed shell or tube (for example, most polychaetes) were excluded from analysis as it is not possible to determine whether these are alive in images. Several spatially blind reviews were conducted to the whole dataset to ensure a robust taxonomic alignment between sites, consisting in side-by side visualization of all specimens classified under the same catalogue label using the ‘Label Review Grid Overview’ tool in BIIGLE^65^; a process that was repeated many times by the same group of expert seabed image scientists, to minimize potential bias^28^. In addition, the likely feeding behaviour (suspension, deposit and scavenger or predator feeding) of each morphotype was inferred from observational knowledge and by reference to similar organisms described in the literature. With a total of 53,512 megafaunal specimens in 13 phyla, our biodiversity dataset was compiled across a wide geographical span (>5,000 km) at the abyss and is of comparable magnitude to the largest datasets that have been used to underpin our theoretical understanding in the deep-sea (for example, refs. ^71,72^).

### Survey design

We compiled invertebrate megafauna occurrences into two different subsets to investigate variations across space in different parameters. These were (1) standing stocks subset (SSdat): containing faunal count data at all taxonomical levels obtained only from scalable still images and thereby associated with a precise measure of the seabed surface area (47,087 specimens); and (2) biodiversity subset (BDdat): containing faunal count data obtained from both image and video surveys but including only specimens identified up to morphotype level (36,432 specimens). Using ArcMap v.6.10 software, we interpolated each specimen across a 10 × 10 km^2^ regional grid to assign and constrain occurrence data to different geographical locations (units of 100 km^2^) within sites. This process divided the SSdat into 23 locations and the BDdat into 28 locations (Fig. 1 and Table 1). Faunal occurrences (or image units, in SSdat) within each geographical location were then randomly resampled without replacement to generate equally sized (in terms of number of specimens encountered) replicate sample units, characterizing the community at each location while minimizing potential spatial autocorrelation biases (for example, ref. ^73^). In locations where surveys covered a large depth spectrum, data were further constrained to 200 m depth bins during replicate sample generation. Following recommendations of minimum sample size for accurate and precise estimation of local megabenthic community features^47^ and to minimize the potential effect of regional gradients in faunal abundance on diversity measures (for example, ref. ^25^), we generated SSdat samples containing 450–500 specimens (taxonomic resolution including specimens identified to morphotype and to higher taxonomical levels) and BDdat samples containing an exact number of 200 specimens (taxonomic resolution including only specimens identified to morphotype level). This process yielded a total of 84 samples standardized for density analyses (standing stocks) and 161 samples standardized for biodiversity analyses (richness and beta-diversity).

### Data analysis

Depth and position (latitude and longitude) of specimens (or images) were averaged within each sample to investigate variations in communities across space and environmental gradients. Sample positions were interpolated with predictions of yearly POC flux (gC_org_ m^−2^ yr^−1^) to the seabed derived by applying vertical flux attenuation^59^ to satellite-derived export flux data (Supplementary Methods). Different ecological metrics were calculated for each sample: (1) standing stocks were assessed as the total numerical abundance of specimens per total unit (m^2^) of seabed surveyed in each SSdat sample; that is, for the whole assemblage, specific populations or faunal groups of interest (taxonomic or functional) and (2) to examine the range of diversity characteristics, Hill’s diversity numbers of order 0 and 1 (ref. ^43^) were calculated in each BDdat sample as morphotype richness (*S*), the exponential form of the Shannon diversity index (exp*H’*), for the whole assemblage.

Preliminary exploration of the interaction between different environmental factors (depth, latitude, longitude and POC flux) across study locations revealed substantial correlations between depth and longitude and also between POC flux and latitude (see Supplementary Fig. 1). Consequently, we choose to investigate only potential interactions between ecological metrics (*y*, that is standing stock and diversity indices) and two main environmental factors (*x*, that is water depth and POC flux, as these were not significantly correlated; see Supplementary Fig. 1). We used the Akaike information criterion ^74^ to select the best fitting linear regression across a range of data transformation types (where ‘exp’ consistently outperformed ‘linear’ and ‘log’).

We followed a simple, data-driven, bottom-up approach to investigate the potential existence of biogeographic boundaries across the depth spectrum of the north Pacific abyss. First, using the whole dataset, we generated ridgeline plots depicting within-group geometric distributions of the total abundance sampled in the most dominant high taxonomic groups (>50 occurrences, minimum taxonomic rank: order). On the basis of the results of this analysis, we assessed the potential variability within and between the megabenthic communities in tentative biogeographical provinces, by grouping samples in three different depth bins: shallow <4,300 m; transition 4,300–4,800 m; deep >4,800 m). Mean values and 95% confidence intervals were calculated for different ecological parameters (faunal density based on SSdat samples and both diversity indices based on BDdat samples) within each tentative province. To contextualize patterns in morphotype richness and the representability of the sampling conducted within each depth range, morphotype accumulation curves were calculated following ref. ^75^, by random resampling of BDdat samples in each depth category 100 times without replacement forming increasingly larger sampling units, using EstimateS v.9.1 software^76^. In addition, using the same methodology, curves were also calculated for study sites encompassing a minimum of three BDdat samples (sampling effort >600 specimens identified to morphotype level).

Variations in community composition across locations, sites and potential biogeographical provinces were further explored using BDdat samples. Dissimilarity in faunal composition between all pairs of samples was calculated using the Bray–Curtis dissimilarity measure on square-root transformed (normalized^77^) taxon abundances. Non-metric multidimensional scaling ordinations were conducted to visualize the rate of dissimilarity (distance) between all pairs of samples; isotropic contour lines depicting broadly approximated depth ranges were fitted using generalized additive models^78^ in the resulting multidimensional scaling plots to aid visualization of beta-diversity patterns across environmental gradients. All data processing and analysis were implemented in R v.4.2.1 (ref. ^79^), using standard analysis methods and functions from the packages: ‘vegan’^80^, ‘AICcmodavg’^81^, ‘ggridges’^82^ and ‘ggplots2’^83^. We report statistical evidence of variations in ecological parameters across environmental gradients (linear regressions, that is *R*^2^ and *P* values) or between biogeographical provinces (95% confidence intervals; significant at *P* < 0.05) using the simplified language of evidence^84^.

### Reporting summary

Further information on research design is available in the Nature Portfolio Reporting Summary linked to this article.

## Supplementary information

Supplementary Information Supplementary Methods, Figs. 1–5 and Tables 1 and 2.

Reporting Summary

## Data Availability

Data generated for this study, invertebrate abyssal megafauna taxa occurrences across the northeast Pacific seafloor, are available at 10.5281/zenodo.7982461 (ref. ^85^). The Abyssal Pacific Seafloor Megafauna Atlas (APSMA image-based taxonomical catalogue) developed to conduct this study is available at 10.5281/zenodo.7765164 (ref. ^66^). Data handling and analyses were implemented using standard methods, software tools and code functions detailed in the Methods.
