# Supplementary material for: Carbonate compensation depth drives abyssal biogeography in the northeast Pacific
Source: Nat Ecol Evol. 2023 Jul 24;7(9):1388–97. doi: 10.1038/s41559-023-02122-9 (PMC10482686; doi:10.1038/s41559-023-02122-9)
Supplement: Supplementary file 1 — Supplementary Methods, Figs. 1–5 and Tables 1 and 2. [file 41559_2023_2122_MOESM1_ESM.pdf]

nature ecology & evolution

Article https://doi.org/10.1038/s41559-023-02122-9

Carbonate compensation depth drives abyssal biogeography in the northeast Pacific

In the format provided by the authors and unedited

Supplementary Information

This file contains supplemental methods, results, tables and figures under the following section headings:

**Additional methods**	2
Original data	2
S. Figure 1	3
**Additional results**	4
S. Figure 2	4
S. Figure 3	5
S. Figure 4	6
S. Figure 5	6
S. Table 1	7
S. Table 2	7
**Suppl. information references**	8

Additional methods

Original seabed image collection data

NORI-D dataset

High resolution vertical-facing seabed images were collected in May-June 2020 using the remotely operated vehicle (ROV) *Kystdesign Supporter 31* from the *Pacific Constructor* during the "WROV Photo Sampling Environmental Survey", a campaign commissioned by TMC, part of the contractor's environmental management surveys. Seabed images were collected within three geographically different areas within the NORI-D area. In each area, the survey design followed a zigzag pattern made up of five 2km transects with random start (e.g. following[1]). Images were collected with an *Imenco Tiger Shark* camera (Canon IXUS130; Field of view in water: vert.= 32°, horiz.= 39°; resolution: 4320x3240 px) vertically mounted on the ROV (Camera settings: shutter speed=1/60s, ISO=100, aperture= F2.8 and focal length= 5mm). The ROV was set to collect images at an altitude of 1.2m and a speed of 0.1m/s, photos were collected every 15 secs. Only every second image was kept for analysis to remove overlap between adjacent images. Images within a 150m buffer zone within the start and end of a transect were removed to minimise potential spatial autocorrelation bias[2], i.e. independent image transects. Only a central 3600x3100 pixel section of each image was retained to remove illumination gradients towards the edges of each frame. At the target altitude, each cropped image covered 2.06 m^2 of seabed. A total of 2500 (out of a final selection of 12282) images randomly selected across the three study locations within NORI-D area were used for analysis in this study. These data were used for both biodiversity and faunal density analyses (see Methods, Table 1).

KIOST dataset (KODOS and APEI-9)

Seabed imagery surveys were carried out during the KODOS1801 and the KODOS1901 expeditions onboard RV Kilo Moana in the Korea contract area KR5 (13°31′–13°03′W, 9°05′–11°40′N) and the APEI9 (127°08′–127°05′W, 10°23′–10°26′N) areas. The KODOS1801 expedition was conducted from March 5 to April 3, 2018 and the KODOS1901 expedition was conducted from March 4 to April 1, 2019. Randomly allocated seabed video transects (lineal extension > 28 km) were obtained using *TowCam* system (SN DSC005) from the Woods Hole Oceanographic Institution[3]. The camera was flown between 2 to 6 m off the bottom with a shutter speed of 1/60. A total of 11,436 invertebrate megafauna specimens > 10 mm were detected across the 4 locations surveyed at the KODOS site and the location at APEI-9. From these, a random subset of 1057 specimens encountered at the APEI-9 (i.e. 3 transects) and 4493 specimens encountered within 2 locations at the KODOS site (i.e. 7 transects) were used in this study. These data were used for biodiversity analyses only (see Methods, Table 1).

Estimation of POC flux to the seafloor

Particulate organic carbon (POC) flux to the seafloor across the abyssal N Pacific was estimated by applying a vertical flux attenuation equation to satellite-derived primary production[4] and sea surface temperature based on the relationship defined in[5]. Flux attenuation described by "Martin's b" parameter[6] was estimated using a collation of deep moored sediment trap data as described in[7]. Seafloor depth was taken from the ETOP01 global relief model[8]. All data were spatially averaged to a 10 x 10 km grid. POC flux to the seafloor (in g C_{org} m^{-2} yr^{-1}) was extracted at each sample location using a nearest neighbour approach.

Assessment of interactions between environmental variables

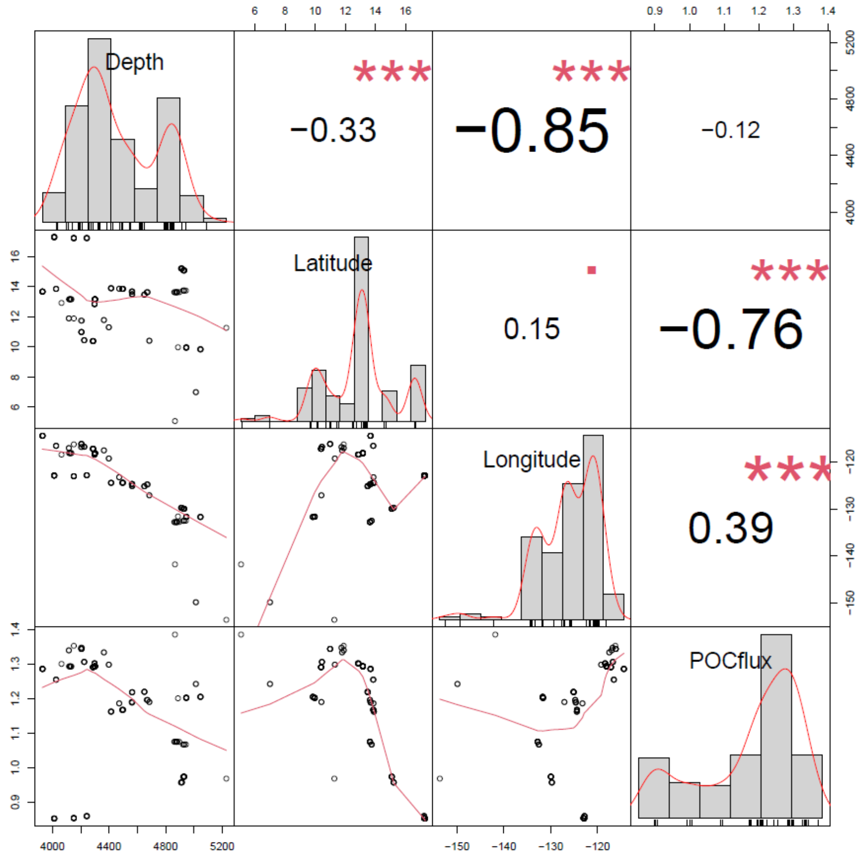

Supplementary Figure 1. Pearson's correlation matrix calculated between environmental variables across the 161 sample locations of this study within the northeast Pacific and results of tests for association between paired samples based on Pearson's product moment correlation coefficient. Red stars = test significance levels; Depth-Latitude (P= 1.7 x 10^{-5}), Depth-Longitude (P= 2.2 x 10^{-16}), Depth-POC flux (P= 0.125), Longitude-Latitude (P= 0.064), Latitude-POC flux (P= 2.3 x 10^{-16}), and Longitude-POC flux (P= 2.4 x 10^{-7}).

Additional results

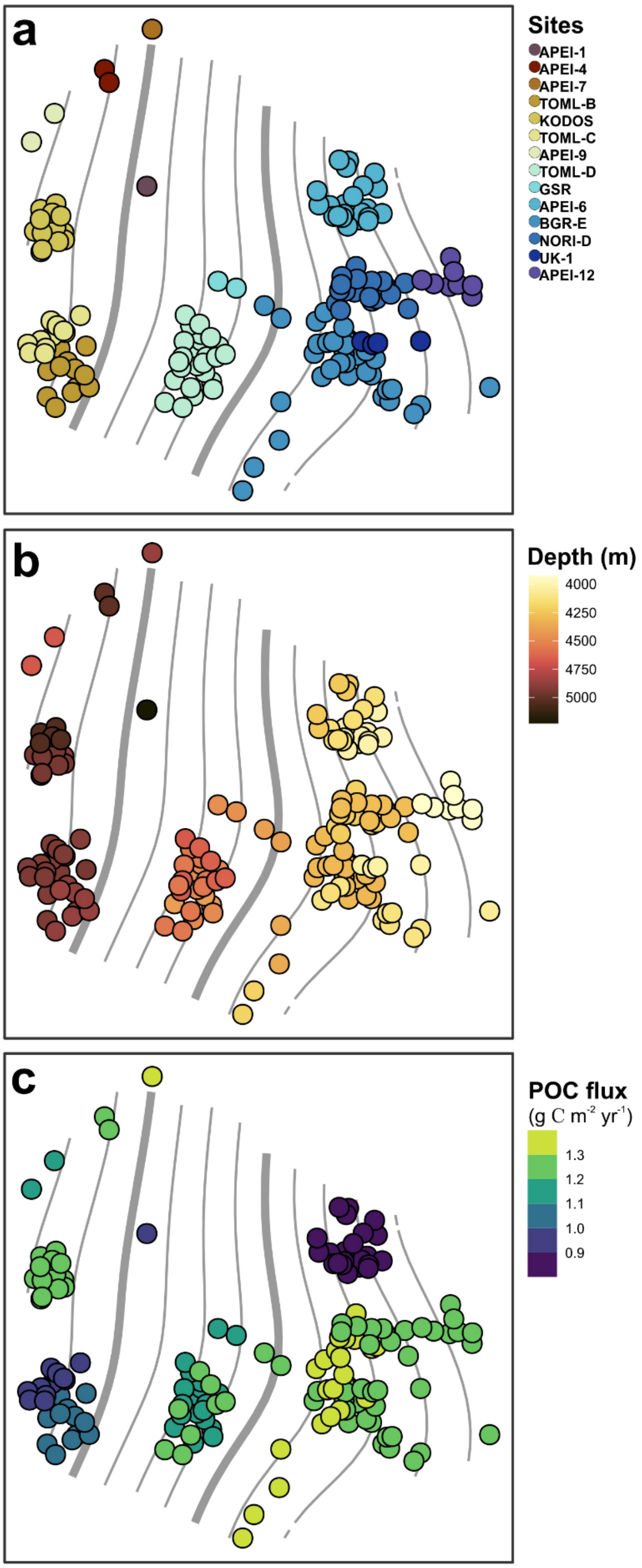

Supplementary Figure 2. MDS plots depicting assemblage dissimilarity between 161 samples (for which circa 200 specimens identified to morphotype level per sample) extending across 28 geographical locations within the NE Pacific. Sample point colour coding: **a)** study site of each sample location; **b)** mean depth at sample location; **c)** estimated rate of POC flux to the seabed at sample location. Isotropic contour lines (fitted using GAMs) represent rough approximates of depth-range bins -to aid visualisation of patterns. Thick lines highlight the range of the carbonate compensation depth (CCD; 4400-4800 m) across the northeast Pacific.

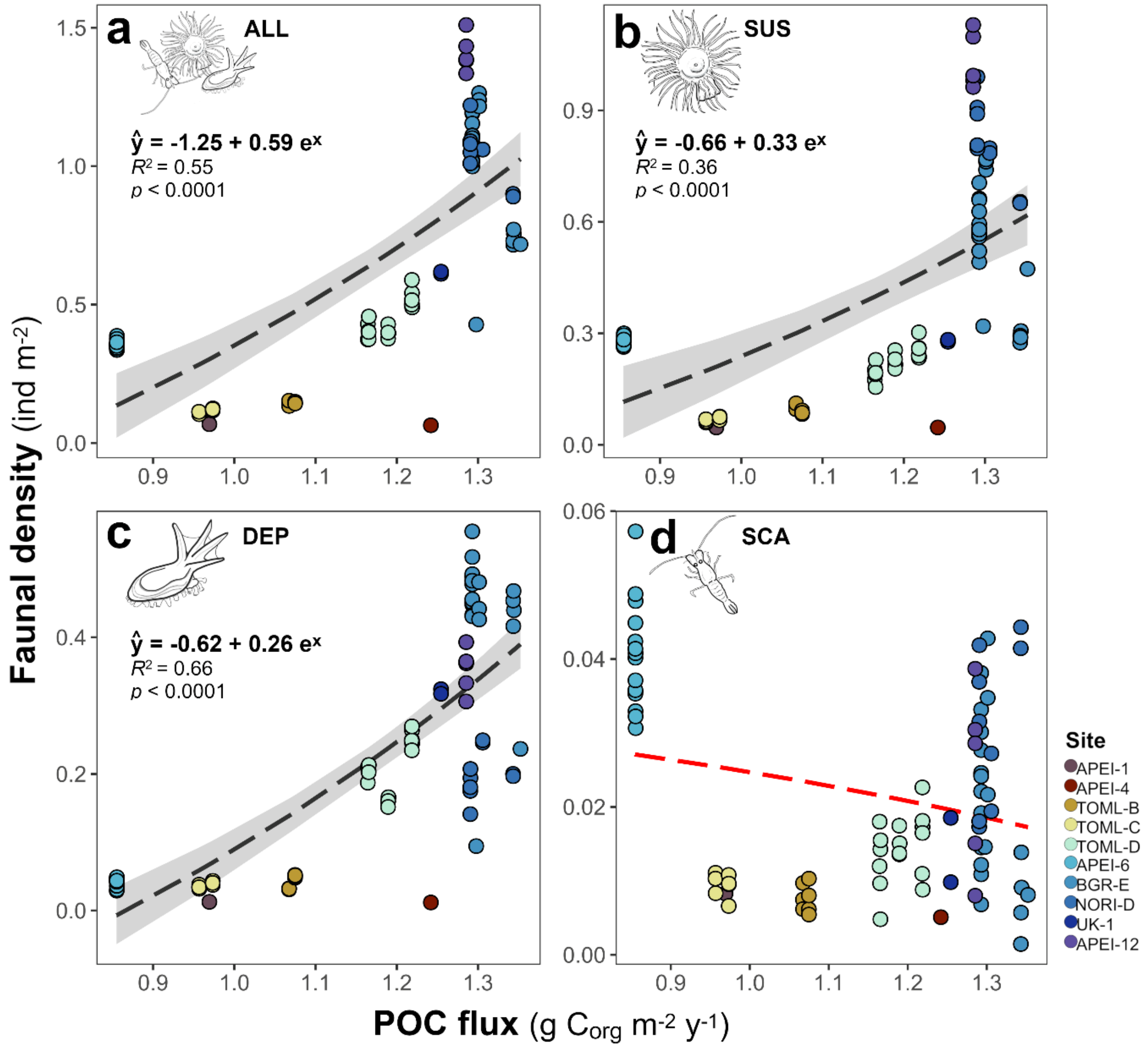

Supplementary Figure 3. Variations in standing stocks across the predicted gradient of particulate organic carbon (POC) flux to the seabed in the northeast Pacific abyss. Faunal densities (individuals m^{-2}) were calculated in 84 independent community samples (containing 400-500 specimens) extending across 23 geographical locations. Values calculated for each independent sample (points) and results of linear regression of density values fitted with an exponential function along the gradient of POC flux; mean (dashed-line) and 95% confidence intervals (shallowing) in statistically significant interactions. **a)** Variations in whole community density ($F_{1,82}$= 101.2, P= 4.99 x 10^{-16}). **b)** Variations in suspension-feeding fauna density ($F_{1,82}$= 46.92, P= 1.19 x 10^{-9}). **c)** Variations in deposit-feeding fauna density ($F_{1,82}$= 153.9, P= 2.2 x 10^{-16}). **d)** Variations in predator and scavenger fauna density ($F_{1,82}$= 5.64, P= 0.019).

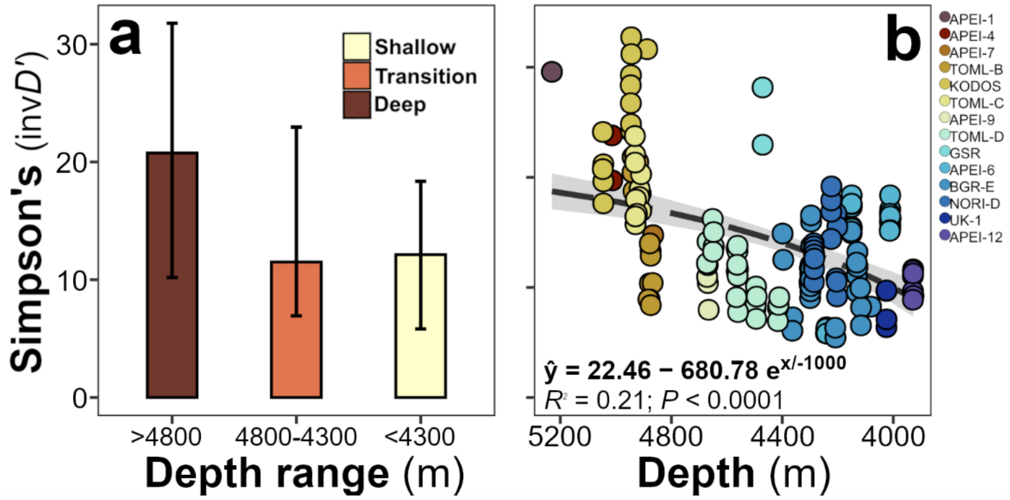

Supplementary Figure 4. Variations in evenness diversity across seabed communities in the northeast Pacific abyss. Estimates of the exponential form of Shannon's diversity index (inv*D'*) calculated in 161 independent community samples (for which circa 200 specimens identified to morphotype level per sample) extending across 28 geographical locations. **a)** Variations between abyssal provinces (n = 81 samples in Shallow, 39 in Transition, and 41 in the Deep province). Mean values (bars) and 95% confidence intervals (error bars) across all the samples in each province. **b)** Variations across the depth range ($F_{1,159}$= 91.07, P= 2.1 x 10^{-16}). Values calculated for each independent sample (points), with results of linear regression depicted; mean (dashed-line) and 95% confidence intervals (shallowing). Note depth was plotted throughout decreasing from left to right, e.g. west to east, to mirror the approximate spatial pattern across the CCZ.

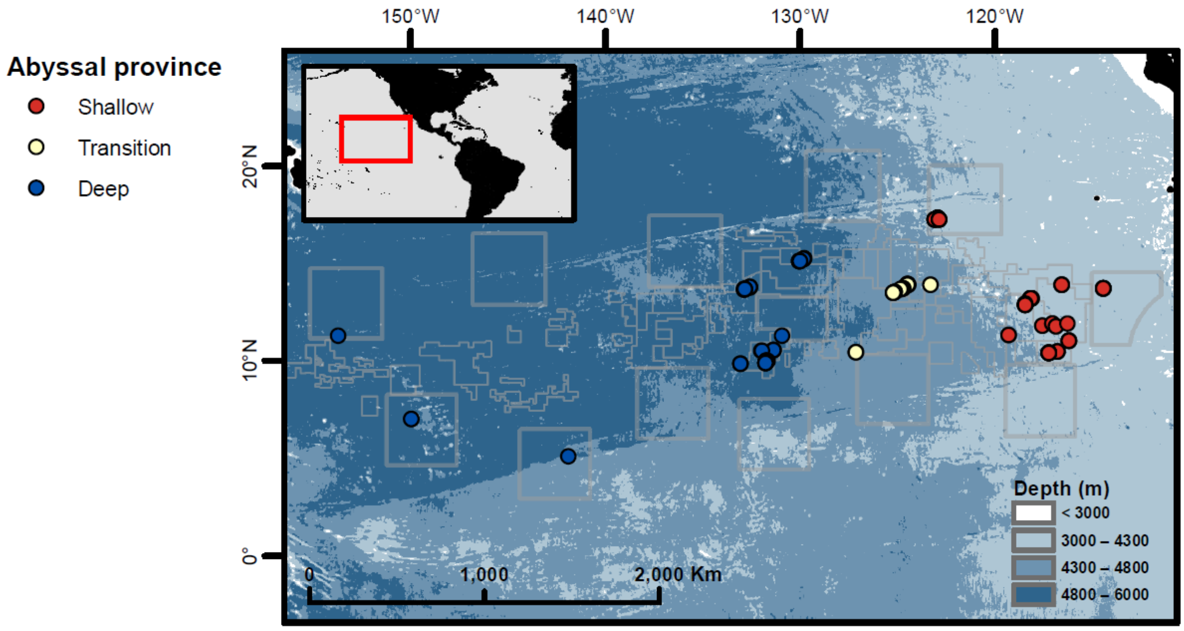

Supplementary Figure 5. Map of study locations surveyed using deep sea robots (ROVs and AUVs) within the CCZ. Points indicate study locations (depths: 3900-5300 m) where data from seabed imagery studies were collated from, aligned and reanalysed using standardised methodology for homogenous detectability and taxonomic identification of invertebrate benthic megafauna (animals > 10 mm). The colour of the points depicts the abyssal province (shallow or deep) of each location based on the results of this study.

Supplementary Table 1. Relative abundance (%) of different taxonomic groups in the deep (> 4800 m depth), transition (4300-4800m depth), and shallow (< 4300 m) provinces across the NE Pacific abyss. Table showing only groups representing at least 1% of the abundance surveyed in any of the different provinces. Total abundance surveyed per province: Deep = 12,254 specimens; Intermediate = 15,526 specimens; Shallow = 25,732 specimens.

Taxonomic group	Deep	Interm.	Shall
Ophiuroidea	5.2	32.3	22.1
Actiniaria	32.3	18.7	7.7
Alcyonacea	0.8	9.1	29.5
Bryozoa	4.0	5.2	11.8
Hexactinellida	13.4	6.5	3.9
Demospongiae	4.0	6.0	6.8
Holothuroidea	13.0	2.7	1.9
Echinoidea	5.2	5.0	2.0
Antipatharia	6.1	3.6	1.3
Polychaeta	5.2	1.9	0.7
Decapoda	2.7	1.8	1.0
Asteroidea	1.7	3.2	2.8
Crinoidea	0.8	0.9	1.3
Bivalvia		0.1	2.1
Polyplacophora		0.2	1.7
Isopoda	1.0	0.4	1.0
Corallimorpharia	1.3	0.2	0.5

Supplementary Table 2. Top 10 most dominant taxa in each abyssal province. Total abundance (incl. only specimens identified up to morphotype taxonomic resolution) surveyed within each province and proportion from the total number of specimens encountered.

	Deep		Transition		Shallow	
Dominance Rank	Taxon	n	Taxon	n	Taxon	n
1	ACT_022	553	OPH_010	1840	OPH_010	2189
2	URC_003	517	URC_003	715	ALC_008	1735
3	ACT_019	413	HEX_021	697	HEX_021	1363
4	ANT_002	401	ACT_022	683	ALC_009	1199
5	ANN_003	393	ANT_002	378	BRY_003	1003
6	BRY_016	366	ACT_004	335	ALC_007	598
7	HEX_015	349	AST_004	307	BRY_001	558
8	HOL_076	341	HEX_015	301	MOL_021	507
9	OPH_010	300	ALC_008	206	MOL_002	427
10	HEX_021	288	ANN_003	175	ALC_004	416
total Top10		3921		5637		9995
total sampled		9336		9516		17580
Top10 %		0.42		0.59		0.57

Supplementary Information references

1 Strindberg, S. & Buckland, S. T. Zigzag survey designs in line transect sampling. *Journal of Agricultural, Biological, and Environmental Statistics* **9**, 443-461, doi:10.1198/108571104x15601 (2004).

2 Legendre, P. Spatial Autocorrelation: Trouble or New Paradigm? *Ecology* **74**, 1659-1673, doi:doi:10.2307/1939924 (1993).

3 Fornari, D. J. & Group, W. T. A new deep-sea towed digital camera and multi-rock coring system. *Eos, Transactions American Geophysical Union* **84**, 69-73, doi:https://doi.org/10.1029/2003EO080001 (2003).

4 Carr, M.-E. Estimation of potential productivity in Eastern Boundary Currents using remote sensing. *Deep Sea Research Part II: Topical Studies in Oceanography* **49**, 59-80, doi:https://doi.org/10.1016/S0967-0645(01)00094-7 (2001).

5 Henson, S. A. *et al.* A reduced estimate of the strength of the ocean's biological carbon pump. *Geophysical Research Letters* **38**, doi:https://doi.org/10.1029/2011GL046735 (2011).

6 Martin, J. H., Knauer, G. A., Karl, D. M. & Broenkow, W. W. VERTEX: carbon cycling in the northeast Pacific. *Deep Sea Research Part A. Oceanographic Research Papers* **34**, 267-285, doi:https://doi.org/10.1016/0198-0149(87)90086-0 (1987).

7 Henson, S. A., Sanders, R. & Madsen, E. Global patterns in efficiency of particulate organic carbon export and transfer to the deep ocean. *Global Biogeochemical Cycles* **26**, doi:10.1029/2011gb004099 (2012).

8 Amante, C. & Eakins, B. W. ETOPO1 arc-minute global relief model : procedures, data sources and analysis. (2009).
