# Supplementary material for: Carbonate compensation depth drives abyssal biogeography in the northeast Pacific
Source: Nat Ecol Evol. 2023 Jul 24;7(9):1388–97. doi: 10.1038/s41559-023-02122-9 (PMC10482686; doi:10.1038/s41559-023-02122-9)
Supplement: Supplementary file 2 — Reporting Summary [file 41559_2023_2122_MOESM2_ESM.pdf]

nature portfolio

Corresponding author(s): Erik Simon-Lledo

Last updated by author(s): May 30, 2023

Reporting Summary

Nature Portfolio wishes to improve the reproducibility of the work that we publish. This form provides structure for consistency and transparency in reporting. For further information on Nature Portfolio policies, see our Editorial Policies and the Editorial Policy Checklist.

Statistics

For all statistical analyses, confirm that the following items are present in the figure legend, table legend, main text, or Methods section.

n/a	Confirmed	
☐	☒	The exact sample size (*n*) for each experimental group/condition, given as a discrete number and unit of measurement
☐	☒	A statement on whether measurements were taken from distinct samples or whether the same sample was measured repeatedly
☐	☒	The statistical test(s) used AND whether they are one- or two-sided<br>*Only common tests should be described solely by name; describe more complex techniques in the Methods section.*
☐	☒	A description of all covariates tested
☐	☒	A description of any assumptions or corrections, such as tests of normality and adjustment for multiple comparisons
☐	☒	A full description of the statistical parameters including central tendency (e.g. means) or other basic estimates (e.g. regression coefficient) AND variation (e.g. standard deviation) or associated estimates of uncertainty (e.g. confidence intervals)
☐	☒	For null hypothesis testing, the test statistic (e.g. *F*, *t*, *r*) with confidence intervals, effect sizes, degrees of freedom and *P* value noted<br>*Give P values as exact values whenever suitable.*
☒	☐	For Bayesian analysis, information on the choice of priors and Markov chain Monte Carlo settings
☒	☐	For hierarchical and complex designs, identification of the appropriate level for tests and full reporting of outcomes
☒	☐	Estimates of effect sizes (e.g. Cohen's *d*, Pearson's *r*), indicating how they were calculated

Our web collection on statistics for biologists contains articles on many of the points above.

Software and code

Policy information about availability of computer code

Data collection	ArcMap v.6.10 and BIIGLE v.2.0 used for image data handling
Data analysis	EstimateS ver .9.1<br>R ver 4.2.1<br>vegan package ver 2.6-2<br>AICcmodavg package ver 2.3-1<br>ggridges package ver 0.5.3<br>ggplots2 package ver 3.3.6

For manuscripts utilizing custom algorithms or software that are central to the research but not yet described in published literature, software must be made available to editors and reviewers. We strongly encourage code deposition in a community repository (e.g. GitHub). See the Nature Portfolio guidelines for submitting code & software for further information.

Data

Policy information about availability of data

All manuscripts must include a data availability statement. This statement should provide the following information, where applicable:

- Accession codes, unique identifiers, or web links for publicly available datasets
- A description of any restrictions on data availability
- For clinical datasets or third party data, please ensure that the statement adheres to our policy

Provide your data availability statement here.

Human research participants

Policy information about studies involving human research participants and Sex and Gender in Research.

Reporting on sex and gender	*Use the terms sex (biological attribute) and gender (shaped by social and cultural circumstances) carefully in order to avoid confusing both terms. Indicate if findings apply to only one sex or gender; describe whether sex and gender were considered in study design whether sex and/or gender was determined based on self-reporting or assigned and methods used. Provide in the source data disaggregated sex and gender data where this information has been collected, and consent has been obtained for sharing of individual-level data; provide overall numbers in this Reporting Summary. Please state if this information has not been collected. Report sex- and gender-based analyses where performed, justify reasons for lack of sex- and gender-based analysis.*
Population characteristics	*Describe the covariate-relevant population characteristics of the human research participants (e.g. age, genotypic information, past and current diagnosis and treatment categories). If you filled out the behavioural & social sciences study design questions and have nothing to add here, write "See above."*
Recruitment	*Describe how participants were recruited. Outline any potential self-selection bias or other biases that may be present and how these are likely to impact results.*
Ethics oversight	*Identify the organization(s) that approved the study protocol.*

Note that full information on the approval of the study protocol must also be provided in the manuscript.

Field-specific reporting

Please select the one below that is the best fit for your research. If you are not sure, read the appropriate sections before making your selection.

☐ Life sciences ☐ Behavioural & social sciences ☒ Ecological, evolutionary & environmental sciences

For a reference copy of the document with all sections, see nature.com/documents/nr-reporting-summary-flat.pdf

Ecological, evolutionary & environmental sciences study design

All studies must disclose on these points even when the disclosure is negative.

Study description	This study aims to determine if trends exist in the spatial distribution of abyssal seafloor populations, and which environmental controls might best explain such changes, for the first time at regional to intermediate scales, using a simple bottom-up analytical approach. To this purpose, invertebrate occurrences obtained from comparable seabed imagery collected in multiple locations (constrained to 10 x 10 km seabed patches) across the CCZ abyssal plain (N Pacific) were compiled from previous studies (including three previously unexplored sites) and reanalyzed using standardized methodology. Randomly generated replicate samples with fixed size (i.e. 200 specimens) were used to characterize and compare abundance, diversity and similarity between communities at different locations and whether these variations exhibited any trends depth of food supply.
Research sample	Invertebrate benthic megafauna (animals > 10 mm) occurrences (i.e. latitude, longitude, and depth) were obtained from detection and identification of these species by reanalysis of seabed imagery collected using comparable methods (e.g. high resolution video and stills cameras mounted on deep sea robots). The standardized methodology used, detailed in the methods, ensured homogeneous animal detectability and subsequent taxonomic identification; robustly aligned across all the images processed. This procedure yield to the identification and geo-referencing of >50,000 specimens (13 Phyla) across 28 geographical locations across the N Pacific abyssal seafloor.
Sampling strategy	Sampling strategy was based on standardized protocols for reliable characterization (i.e. min sample size: 200 morphotype-level-identified specimens per community sample) of abyssal benthic megafauna assemblages in seafloor spatial ecology by Simon-Lledo et al 2019a
Data collection	Seabed imagery were collected in multiple expeditions, but the study only used those image datasets that complied with the following conditions (aimed to ensure that image quality enabled a consistent specimen > 10 mm detectability throughout the

	dataset): i) imagery collected between 1–4 m above the seabed; ii) well-lit and high resolution (i.e. minimum resolution at 2 m above-seabed: 1280 x 720 px); iii) total seabed survey area imaged per location >2000 square meters; iv) no overlapping frames included; v) scalable stills collected vertically-facing the seabed (for density analysis only); vi) collected in abyssal nodule field habitats within the CCZ
Timing and spatial scale	Specimen occurrence data were obtained during different deep-sea expeditions conducted between 2010 and 2020 across the 5000 km span of CCZ region
Data exclusions	Taxa living in a closed shell or tube (e.g. most polychaetes) were excluded from analyses as it is not possible to determine whether these are alive in images. Giant seabed foraminifera (Xenophyophores) were also excluded from analysis, for the same reason.
Reproducibility	The original dataset generated and used to run the analyses is provided as as source data files, the software and standard code functions used are all open-sourced, and the taxonomic identification catalogue used to standardize specimen identifications is available on-line.
Randomization	Replicate seabed community samples of fixed size were generated by random selection (without replacement) of specimen occurrences falling within each spatially restricted (i.e. 10 x 10 km patch size) locations.
Blinding	We used BIIGLE 2.0 to blind the location of the images containing each of the specimen occurrences during image re-analysis to minimize potential observer-bias. Several spatially-blind reviews were conducted to the whole dataset to ensure a robust taxonomic alignment between data from different sites, consisting in side-by side visualization of all specimens classified under the same catalogue label using the 'Label Review Grid Overview' tool in BIIGLE 2.0.

Did the study involve field work? ☐ Yes ☒ No

Reporting for specific materials, systems and methods

We require information from authors about some types of materials, experimental systems and methods used in many studies. Here, indicate whether each material, system or method listed is relevant to your study. If you are not sure if a list item applies to your research, read the appropriate section before selecting a response.

Materials & experimental systems

n/a	Involved in the study	
☒	☐	Antibodies
☒	☐	Eukaryotic cell lines
☒	☐	Palaeontology and archaeology
☒	☐	Animals and other organisms
☒	☐	Clinical data
☒	☐	Dual use research of concern

Methods

n/a	Involved in the study	
☒	☐	ChIP-seq
☒	☐	Flow cytometry
☒	☐	MRI-based neuroimaging
